# Intranasal Perillyl Alcohol for Glioma Therapy: Molecular Mechanisms and Clinical Development

**DOI:** 10.3390/ijms19123905

**Published:** 2018-12-06

**Authors:** Thomas C. Chen, Clovis O. da Fonseca, Axel H. Schönthal

**Affiliations:** 1 Department of Neurological Surgery, Keck School of Medicine, University of Southern California, Los Angeles, CA 90089, USA; 2 Department of General and Specialized Surgery, Antonio Pedro University Hospital, Fluminense Federal University, Niterói, RJ 24220, Brazil; clovis.orlando@uol.com.br; 3 Department of Molecular Microbiology and Immunology, Keck School of Medicine, University of Southern California, Los Angeles, CA 90089, USA

**Keywords:** intranasal, perillyl alcohol, temozolomide

## Abstract

Intracranial malignancies, such as primary brain cancers and brain-localized metastases derived from peripheral cancers, are particularly difficult to treat with therapeutic agents, because the blood-brain barrier (BBB) effectively minimizes brain entry of the vast majority of agents arriving from the systemic circulation. Intranasal administration of cancer drugs has the potential to reach the brain via direct nose-to-brain transport, thereby circumventing the obstacle posed by the BBB. However, in the field of cancer therapy, there is a paucity of studies reporting positive results with this type of approach. A remarkable exception is the natural compound perillyl alcohol (POH). Its potent anticancer activity was convincingly established in preclinical studies, but it nonetheless failed in subsequent clinical trials, where it was given orally and displayed hard-to-tolerate gastrointestinal side effects. Intriguingly, when switched to intranasal delivery, POH yielded highly promising activity in recurrent glioma patients and was well tolerated. As of 2018, POH is the only intranasally delivered compound in the field of cancer therapy (outside of cancer pain) that has advanced to active clinical trials. In the following, we will introduce this compound, summarize its molecular mechanisms of action, and present the latest data on its clinical evaluation as an intranasally administered agent for glioma.

## 1. Introduction

Intracranial malignancies consist of a wide spectrum of different tumor types with particular challenges to treatment and generally poor prognosis. Among the most common primary brain tumors are stage III astrocytoma and stage IV glioblastoma (GB) in adults, and medulloblastoma in pediatric patients. Other primary brain tumor types, such as meningiomas and pituitary adenomas, are oftentimes benign, but can progress to an aggressive phenotype. In comparison to these primary brain malignancies, intracranial metastases derived from systemic cancers of the lung, breast, skin, gastrointestinal tract and kidney are much more prevalent. In the United States, nearly 200,000 patients are newly diagnosed with such secondary brain tumors each year [1,2,3].

The past 30 years have seen substantial progress in brain imaging, advances in surgical technologies and intervention, availability of chemotherapies and targeted drugs, and emergence of multimodality treatment regimens that have reduced morbidity and prolonged survival of afflicted patients. Yet, most patients diagnosed with intracranial malignancies still die of the disease. In the case of GB, prognosis is particularly poor. Current standard of care consists of surgery (when safely feasible), followed by radiotherapy (RT) with concomitant and adjuvant temozolomide (commonly called the ‘Stupp protocol’), which was shown to result in median survival of about 15 months [4,5]. Very recently, the alternating electric fields generator Novo/TTF (tumor treating fields), a type of electromagnetic field therapy that uses low-intensity electrical fields, was approved for GB and added to the available treatment options [6]. In a phase 3 clinical trial with newly diagnosed GB patients, it extended median overall survival by another 5 months over the Stupp protocol, to about 21 months [7]. However, its more widespread application is limited by very high cost and reluctance of some health insurance carriers to cover this treatment [8].

The above-mentioned timelines for overall patient survival have to be viewed in the context of clinical trials. It is important to note that overall health of many GB patients may not be robust enough for participation in clinical trials, nor sufficient to receive all aspects of current standard of care (surgery, RT, temozolomide). As a result, median survival of the entirety of GB patients seen in routine clinical practice is significantly less than what is reported for those treated with the Stupp protocol or with Novo/TTF. For example, before 2005 (sometimes referred to as the pre-temozolomide era), median survival of GB patients was 8.1 months, based on an analysis [9] of data from 6,673 patients collected by the Surveillance, Epidemiology, and End Results (SEERT) Program [10] of the National Cancer Institute of the U.S. National Institutes of Health during the years 2000–2003. A subsequent analysis of SEERT Program data from 7259 patients diagnosed during the early years of the temozolomide era (2005–2008) revealed median survival of 9.7 months [9]. Now, with some patients having access to Novo/TTF, one might estimate that overall survival perhaps approaches 1 year. Continuing dismal prognosis remains to be driven mostly by elderly patients who often are too frail for extensive treatment regimens [9]. 

In recent years, immunotherapy has garnered substantial attention as a new pillar of cancer treatment [11,12]. Besides immune checkpoint-inhibitory approaches [13,14], numerous therapeutic vaccines [15,16] aimed at CNS primary and metastatic disease are at various stages of development. For example, SurVaxM is being developed as a GB vaccine aimed at survivin, a protein that is overexpressed in many tumor types. The K27M peptide vaccine is aimed at this particular mutation in histone 3 variant proteins. Heat shock protein peptide complex 96 (HSPPC-96) is a vaccine where different peptides are bound to a 96 kD chaperone protein (HSP-96) derived from autologous GB tissue that was resected from the patient to be treated [17]. Rindopepimut, a peptide vaccine aimed at the EGFR deletion mutation EGFRvIII [18,19] seemed particularly promising for vaccine development, because this specific mutation is highly prevalent in about 20–30% of all GBs [20]. Despite encouraging results from early clinical trials, however, a recently completed phase III trial of rindopepimut with temozolomide for patients with newly diagnosed, EGFRvIII-expressing GB failed to yield increased survival of these patients [21]. 

Another, highly personalized, approach toward GB vaccination is based on the patient’s mutanome [22]; it utilizes a mix of peptides, representing personal neoantigens identified by DNA sequencing of the patient’s GB tumor, which may be administered in combination with poly ICLC (polyinosinic-polycytidylic acid stabilized with polylysine and carboxymethylcellulose) for overall greater immune stimulation [23]. In contrast, another therapeutic GB vaccine, ERC1671, does not attempt to identify tumor-specific mutations in a patient; rather, it represents the antipode of the highly personalized, narrowly targeted vaccination approach. It is based on a multi-modular approach, where syngeneic and allogeneic components are mixed and administered to recurrent GB patients [16]. During the course of vaccination, tumor cells derived from the patient to be treated, as well as from three additional, unrelated GB tissue donors, are mixed and subcutaneously injected, either as inactivated cells or cell lysates. This vaccination strategy is expected to trigger an immune response against a broad array of tumor antigens, and preliminary reports have indicated encouraging results [24,25,26].

Despite all encouraging advances in the area of immunotherapy and therapeutic vaccinations, it is accepted that surgery, RT and temozolomide have remained the mainstay of malignant glioma therapy [27,28]. In addition, the blood-brain barrier (BBB), which prevents effective brain entry of most therapeutic agents, continues to constitute a significant obstacle against effective therapy [29]. Although there are indications that barrier function is compromised within brain tumor lesions, it still remains sufficiently intact to significantly impair drug delivery [30,31,32]. For example, a detailed analysis of 2000 individual lesions of breast-to-brain metastases in mice demonstrated great heterogeneity between individual lesions—and even within the same lesion—with regard to BBB permeability; the majority of these lesions displayed inadequate drug uptake through their BBB [30]. A recent critical assessment of existing clinical data from GB patients concluded that “all GB patients have tumor regions with an intact BBB, and cure for GB will only be possible if these regions of tumor are adequately treated” [31].

Different strategies are being pursued to overcome the BBB obstacle. Some of these efforts focus on the disruption of barrier function, for example with intracarotid injections of mannitol [33]. Others seek to modify the physicochemical properties of drug candidates, for instance by means of in silico modeling, to increase the drug’s ability to penetrate an intact BBB [34,35]. Yet another strategy seeks to avoid the BBB altogether, by exploiting direct nose-to-brain transport after intranasal delivery of therapeutic agents [36,37], which will be the focus of this review. 

Perillyl alcohol (POH), a naturally occurring monoterpene, represents the only intranasally delivered anticancer compound that has advanced to clinical phase 1/2 trials. In the following, we will introduce this compound, summarize its molecular mechanisms of action, and present the latest data on its clinical evaluation as an intranasally administered agent for glioma. To fully convey the potential and challenges of this treatment strategy, it is necessary to also introduce the anatomical structures of the nasal cavity, as well as physiologic drug transport mechanisms for brain delivery. However, due to the availability of many excellent reviews on these latter topics, we will keep these respective sections brief.

## 2. Anatomy of the Nasal Cavity 

The nasal cavity (nasal passage, nasal fossa) is separated by the nasal septum into two independent compartments, where each one is divided into three main parts: vestibule (anterior external opening), respiratory region (lined by ciliated epithelium and mucus-secreting goblet cells), and olfactory region (lined by olfactory cells). The cilia of the respiratory mucosa perform a cleaning function by moving mucus and entrapped particulate matter to the pharynx and esophagus for delivery into the stomach. This function plays a critical role in mediating innate immunity and prevents allergens and microorganisms from spreading throughout the body [38,39]. In the context of nasal drug delivery, this same clearing function requires careful consideration, because general disposition of drug across the mucosa may favor intestinal delivery [40,41]. 

Air enters through the nostril, passes the vestibule and nasal valves, and enters the main nasal chamber, which is sectioned by inferior, middle, and superior turbinates (conchae). Due to their curved, narrow morphology, the turbinates substantially increase the overall surface area within the nasal cavity. They regulate inhaled air pressure and control the passage of air flow through the nasal cavity, thereby performing a variety of functions that include filtration, warming and humidification of inhaled air [38,39]. In the context of nasal drug delivery, in particular when sprays are being used, details of air flow are decisive for drug distribution within the nasal cavity [42]. 

The nasal sub-mucosa is extensively vascularized with overall vigorous blood flow. The veins are unusually thin-walled, which supports warming the air entering the nasal passages—and also facilitates drug uptake by the nasal vasculature [43,44]. 

**Figure 1:**
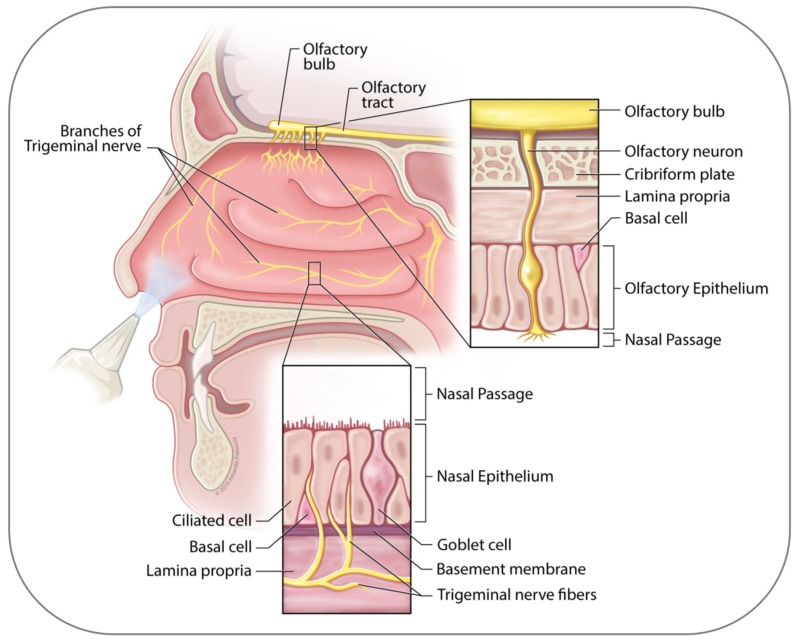
Lateral section of the human nose. Shown are key anatomical structures involved in drug transport from the nose to the brain. Location of olfactory and trigeminal nerves in yellow. Details of olfactory and respiratory epithelia in enlarged sections. See text for details. (Artwork by Amanda Frataccia; © 2018 Amanda Frataccia, provided under CC BY-NC-ND 4.0.).

Innervation of the nasal mucosa involves the olfactory nerve, which controls the sense of smell; branches of the trigeminus (V1 ophthalmic nerve and V2 maxillary nerve), which provide sensory information; autonomic fibers, which determine vasoconstriction and gland secretion; and motor nerves. For considerations of nose to brain drug transport, the olfactory and large trigeminal nerves are of prime relevance (Figure 1) [45,46,47,48]. The sensory fibers of the olfactory bulb are physically exposed to the environment and therefore can come in direct contact to intranasal drugs. 

## 3. Nose to Brain Transport

Intranasal drug delivery combined with nose to brain transport has several advantages over other means of brain-targeted drug administration. It is non-invasive and easy to perform, usually by the patients themselves in the convenience of their home, for example with the use of nasal sprays and nebulizers. Compared to oral drug delivery, it avoids first-pass metabolism by liver enzymes, and brain entry is not limited by the BBB, supporting increased bioavailability and rapid onset of drug action. In comparison, other means of direct drug delivery to the brain harbor substantial drawbacks and risks for the patient. For instance, interventional catheters or placement of drug-laced wafers are invasive procedures that are inconvenient, expensive, and fraught with complications [49]. Similarly, opening of the BBB via intracarotid injection of mannitol, which represents the main clinical method to break down this barrier, carries the risk of seizures, brain embolism, and catastrophic bleeds, and its use is restricted to specialized centers and not widely available [33,50]. 

Some details of the mechanisms of direct nose to brain transport of pharmaceutical agents remain to be characterized, but current models agree that a combination of different routes achieves efficient brain entry [36,37,41,51,52]. The individual contributions of these pathways depend greatly on specific physiochemical and physicochemical properties of each drug, drug formulation and solvents, the respective method used to deliver the drug into the nasal cavity, and the current physiological/pathological status of the nasal region (congestion, runny nose, deformities, trauma, etc.) [44].

**Figure 2:**
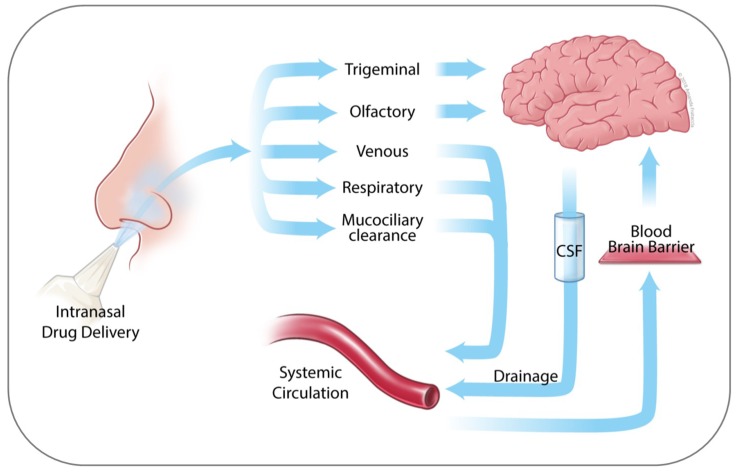
Pathways available to drugs for potential transport to the brain. Trigeminal and olfactory nerves offer direct nose-to-brain transport options. Venous and respiratory pathways, as well as mucociliary clearance, deliver drugs into the systemic circulation, where brain uptake is limited by BBB function. Clearance of drugs from the brain is achieved by several mechanisms, including drainage from the CSF, metabolism by brain-localized cytochrome P450 enzymes, and passing into the systemic circulation [55,56]. (Artwork by Amanda Frataccia; © 2018 Amanda Frataccia, provided under CC BY-NC-ND 4.0.).

As outlined in Figure 2 (and extensively reviewed in several recent reviews [36,37,41,51,52,53]), the main direct pathways from nose to brain are neuronal and primarily consist of olfactory nerve (via olfactory bulb) and respiratory epithelium/trigeminal branches (to lamina propria and brain stem). Other, non-direct pathways involve drug transport into the lungs (especially during inhalational drug administration), uptake into nasal veins, and mucociliary clearance with postnasal drip into the stomach, all of which distribute the drug into the systemic circulation where it encounters the BBB as a decisive obstacle. In the vast majority of cases, drugs are poorly BBB permeable [54], and for that reason these latter pathways play only minor to no roles in drug brain delivery, instead further relying on neuronal transport. 

The process of neuronal drug transport involves two parallel mechanisms: slow intracellular and more expedient extracellular movement. Intracellular transport begins with uptake of the drug molecule by an exposed neuron, trafficking of the endocytic vesicle in the cytoplasm toward the neuron’s projection site, followed by release through exocytosis. The extracellular (paracellular) mechanisms starts with drug entering into perineural space, followed by transport along the neuronal axon into the brain [36,37,45].

## 4. Perillyl Alcohol

In the field of intranasal drug delivery for cancer therapeutic purposes, no compound has been more widely studied than POH ((*S*)-(–)-perillyl alcohol), which has been administered to hundreds of glioma patients over many years. It is also known as perilla alcohol, perillol, isocarveol, p-metha1,7-diene-6-ol, or 4-isopropenylcyclohexenecarbinol, and its IUPAC name is 4-(prop-1-en-2-yl)cyclohex-1-en-1-yl methanol.

POH is a natural compound belonging to the group of monocyclic terpenes found in the essential oils of various botanicals, including citrus fruits, peppermint, spearmint, cherries, cranberries, sage, lemongrass, lavender, celery seeds and others [57]. It is a metabolite of limonene and thus derived from the mevalonate/isoprenoid pathway. After administration to humans or other mammals, POH undergoes stepwise oxidation to perillyl aldehyde, perillic acid and dihydroperillic acid, followed by glucuronidation and subsequent excretion primarily via the renal system [58,59,60]. Medicinal interest in this natural compound received an enormous boost during the final decade of the previous century, when it was discovered that POH exerted potent anticancer activity in cell culture and rodent tumor models. 

### 4.1. Molecular Mechanisms of POH Function

Although often labeled a Ras inhibitor, the molecular and cellular impact of POH is pleiotropic and affects multiple cellular targets and growth-regulatory processes. For instance, POH has been shown to impact the activity of components of the cell cycle machinery. Exposure of cancer cells to POH in vitro resulted in increased expression of p15 (*INK4b*), p21 (*WAF1/Cip1*), and p27 (*Kip1*), all of which belong to the group of cyclin-dependent kinase (CDK) inhibitors that serve to arrest cell cycle progression [61,62,63,64]. Conversely, POH was able to down-regulate expression of several cyclin proteins, which are regulatory subunits of CDKs and essential for their kinase function [62,63,64,65]. It was also found to down-regulate p34(cdc2) (also called cdk1), which is the key CDK essential for entry and progression through M phase [66]. As a result, potent cell cycle arrest ensued, which was followed by apoptosis in the large variety of cancer cell lines that were investigated [55,61,62,65,67,68,69,70]. 

POH was also shown to impact other cellular proteins involved in cell growth control, such as the immediate-early gene products c-Fos and c-Jun [64,71], which heterodimerize to form transcription factor AP-1. Although AP-1 harbors pro-oncogenic properties, it can also be part of the c-Jun N-terminal kinase (JNK)/stress-activated protein kinase pathway that negatively impacts cell proliferation in response to POH [72]. Similarly, POH is a potent trigger of endoplasmic reticulum (ER) stress, as evidenced by potent induction of GRP78 (glucose-regulated protein of molecular weight 78 kDa) and CHOP (CCAAT/enhancer-binding protein homologous protein) [73]. The ER stress response features a dichotomy of antagonizing outputs, exemplified by pro-survival GRP78 and pro-apoptotic CHOP, which constitute critical components of this system that wrestle for dominance. The eventual outcome generally is dependent on the severity of the stressful insult; in this case, the extent of POH exposure. 

Telomerase represents yet another recognized target of POH. Treatment of prostate cancer cells with biologically relevant concentrations of the monoterpene resulted in increased turnover of the telomerase catalytic subunit reverse transcriptase (hTERT), without significant effect on its mRNA levels [74]. Further analysis revealed that hTERT inhibition involved disruption of a large complex of proteins, consisting of hTERT, mTOR (mammalian target of rapamycin), Raptor, HSP90 (heat shock protein of molecular weight 90 kDa), and S6K (ribosomal S6 kinase) [75]. In addition, POH caused dephosphorylation of 4E-BP1 (eIF4E-binding protein) and disrupted interactions between eIF4E (eukaryotic initiation factor 4E) and eIF4G, which are key components of the translational cap binding complex [76,77]. Combined, these studies provided intriguing examples of translational and post-translational effects of POH. 

Further cellular targets of POH include Na/K-ATPase (sodium/potassium adenosine triphosphatase), Notch, NF-κB (nuclear factor kappa B), and TGFβ (transforming growth factor beta). Na/K-ATPase is of relevance, because it may be overexpressed in certain cancer types, and there are indications that its inhibition by POH may be linked to the pro-apoptotic function of the JNK stress pathway [72,78]. The Notch signaling pathway plays a role in tumor cell invasion and metastasis, and it was recently demonstrated that POH could inhibit liver cancer cell migration and invasion in vitro, which was achieved via inhibition of the Notch pathway [79]. Regarding NF-κB, investigators demonstrated that POH treatment of cancer cells caused a persistent decrease of calcium levels, which resulted in inhibition of a calcium-dependent, constitutive NF-κB pathway, eventually leading to apoptosis [80]. In the case of TGFβ, it was shown that POH treatment of tumor-bearing mice stimulated activity of this pathway in their mammary tumors, which correlated with tumor regression in vivo [64,81,82]. Besides providing insight into molecular pathways that are impacted by POH, these in vivo studies with mouse tumor models clearly demonstrated anti-tumor activity of monoterpenes in vivo. Several other in vivo studies validated the anticancer potency of POH in various rodent models of breast, liver, colorectal, and pancreatic cancer [55,63,83,84,85].

While it is conceivable that most, perhaps all, molecular and cellular effects of POH take place in brain tumor cells as well, there is only a limited number of studies in this regard. For example, POH was shown to induce apoptosis in established glioblastoma cell lines, including drug-resistant variants, as well as in a culture of primary patient GB cells, and these effects involved aggravation of ER stress, as indicated by increased expression of GRP78 and CHOP [67,73,86]. Inhibition of Na/K-ATPase, with critical involvement of the JNK stress pathway leading to apoptosis, has also been thoroughly documented in different GB cell lines in vitro [72,78]. At the cellular level, POH consistently inhibited invasion and migration of glioma cells in scratch assays, Boyden chamber studies, and the chicken chorioallantoic membrane (CAM) metastasis model [86,87]. This latter finding is of particular interest in view of the aggressive, infiltrative nature of malignant glioma noted in the clinic [88]; it indicates that POH perhaps might be able to exert a two-pronged therapeutic attack against glioma, involving not only pro-apoptotic mechanisms, but migration/invasion-inhibitory effects as well. While this hypothesis awaits validation, a study by Cho et al. [73] demonstrated in vivo therapeutic activity of POH in an orthotopic mouse glioma model. Notably, POH was administered via intranasal delivery, and the positive results contributed to the design of a clinical study in GB patients (see details below).

### 4.2. Role of Ras Pathway in POH Function

POH is frequently referred to as a Ras inhibitor. However, its impact on the Ras pathway has not been completely clarified, and different studies have generated conflicting outcomes. The earliest studies on this topic showed that POH could inhibit the activity of geranylgeranyl-protein transferases (GGPTases) and farnesyl-protein transferase (FPTase), which are enzymes of the mevalonate pathway, generating isoprenoids for posttranslational modifications of proteins [89,90,91,92,93]. Posttranslational prenylation, in particular farnesylation, is required for membrane association and activity of all three (H, K, N) Ras protein isoforms, both during normal function as well as for their transforming capacity [94,95]. Based on the negative impact of POH on key enzymes of the mevalonate pathway, it was postulated that this monoterpene might exert its anticancer effects via blockage of these proteins’ required posttranslational modifications [96,97].

However, several independent studies presented results that are not consistent with the proposed model that posttranslational inhibition of Ras plays an important role in mediating the growth-inhibitory and apoptosis-inducing effects of POH. For example, the concentrations of POH that are required to inhibit GGPTase and FPTase activity are generally higher than those that impact cell proliferation and cell survival [91,98,99]. Furthermore, while a decrease in farnesylated Ras protein indeed was observed after treatment of cells with POH, this decrease appeared to be due to reduced de novo synthesis of the protein, rather than a blockage of posttranslational modification [87,99,100,101,102].

The downstream pathways of Ras signaling involve the MAPK (mitogen-activated protein kinase) pathway, with ERK (extracellular signal-regulated kinase) as a key component. Therefore, ERK activation has been used as a readout of active Ras signaling. In one study, with POH, no difference in ERK activity could be documented after treatment of cells with the monoterpene [103]. In another study, POH did in fact cause decreased ERK activity, which closely correlated with inhibition of POH-induced cell growth; however, further dissection of this effect revealed that ERK inhibition in this case was achieved in an entirely Ras-independent fashion [104]. Further studies also showed decreased ERK activity of POH-treated cells, which coincided with significant decreases in total amount of Ras protein, rather than specific posttranslational Ras modification [87,105]. Using ^35^S-methionine pulse-labeling, Hohl et al. [102] demonstrated that lower amounts of farnesylated Ras protein after POH treatment were due to reduced synthesis of new Ras protein, rather than specific inhibition of Ras posttranslational modification. 

On the other hand, a few studies presented outcomes that were actually consistent with posttranslational inhibition of Ras activity by POH. For instance, using a mouse model of phorbol ester-induced skin tumors, Chaudhary et al. [106] showed that TPA (12-O-tetradecanoyl phorbol-13-acetate) promoted skin tumor development that correlated with substantially increased amount of membrane-bound Ras, along with elevated levels of ERK activation in their tumor tissue. When these mice were treated with POH, their tumors displayed much less membrane-bound Ras, but increased amount of cytosolic Ras, and reduced ERK activity, indicating POH-mediated posttranslational down-regulation of Ras signaling activity [106]. A separate study in pancreatic tumor cells indicated that POH might have isoform-specific effects on Ras [56]. These authors showed that POH inhibited farnesylation of H-Ras, but not K-Ras, which correlated with down-regulation of MAPK pathway signaling. This isoform-specific effect is intriguing, because it is known that K-Ras and N-Ras, but not H-Ras, are subject to alternative posttranslational modification in response to FPTase inhibition [95].

Altogether, the sum of the above studies provide pros and cons toward the popular model of POH as a posttranslational Ras inhibitor by virtue of its interference with the mevalonate pathway. At present, the majority of studies indicate that this model is not generally applicable, but that it may potentially be isoform-specific and dependent on distinct cell growth conditions. In comparison, inhibition of overall Ras protein synthesis, and related small-molecule GTPases [87,99,107,108], by POH appears well documented, although this level of regulation seems independent of the monoterpene’s impact on the mevalonate pathway. Rather, it adds Ras to the general list of protein targets that are presented in Section 4.1 above.

### 4.3. Clinical Testing of Oral POH

By the turn of the millennium, preclinical studies of POH had thoroughly established its anticancer potency in a variety of in vitro and in vivo tumor models. Consequently, several clinical phase 1 and 2 studies were under way, which investigated safety and activity of oral POH in cancer patients (see detailed references in [109]). POH was formulated in gelatin capsules and, due to its short biological half-life, was administered 3–4 times daily on a continuous basis over several months. Initial phase 1 trials used 2.1 to 2.4 g/m^2^ POH delivered in 3 doses [110,111], or 4.8 g/m^2^ delivered in 4 doses [59], and eventually the MTD (maximum tolerated dose) was established at 8.4 g/m^2^ per day, delivered in four equal doses distributed over the day [112]. For most subsequent phase 2 trials, 4.8 g/m^2^, distributed over 4 doses, was used [113,114,115,116,117], although in some of these trials POH was dose-escalated to 6.0 and 6.4 g/m^2^, respectively, for patients who tolerated the initial dose [113,114,116] (Table 1). 

Despite great effort and mostly good patient compliance in these clinical studies, therapeutic activity of POH was mostly disappointing. In general, toxicity was mild to moderate, and predominantly entailed gastrointestinal (GI) effects and fatigue, although there was significant heterogeneity in tolerability of POH [58,59,113]. In most trials, POH was supplied as 500-mg capsules that contained 250 mg POH along with 250 mg soybean oil, which meant that patients had to swallow a large number of capsules multiple times a day (35–50 total per day in phase 2 trials) over periods of many months [114]. While GI toxicity was not severe, the continuing dosing nonetheless resulted in unrelenting, chronic nausea, belching, reflux, diarrhea or constipation, and fatigue, which were difficult to endure and in a few cases resulted in patients withdrawing their participation from the trials [114,115,116].

Based on unimpressive clinical activity, in combination with the unremitting nature of hard-to-tolerate GI effects and fatigue, oral POH did not advance to phase 3 clinical trials and therefore has not entered clinical practice for indications of cancer treatment. Despite this disappointment, some hope remains, as it appears that changing delivery from oral to intranasal might be able to resurrect this monoterpene’s potential as an anticancer agent. 

### 4.4. Intranasal POH

A case study published in 2006 [118] was the first to describe intranasal application of POH to a cancer patient. It reported the case of a 62-year-old woman with anaplastic oligodendroglioma, who presented with repeat recurrence, despite combination treatment with surgery and radio-chemotherapy. After giving intranasal 0.3% concentration POH four times daily over several months, an MRI scan revealed reduction of the enhancing lesion, indicating tumor shrinkage while undergoing POH inhalational therapy [118]. Since then, results from clinical phase 1 and 2 trials in Brazil have further supported the anticancer potential of intranasal POH. The first one included 37 patients with recurrent malignant glioma, and 0.3% POH was administered 4 times per day at 55 mg per dose (220 mg/day) [119]. It was reported that this novel treatment strategy resulted in decreased tumor sizes and increased survival in several patients. At the same time, it was very well tolerated; there were no reports of toxicity, even in patients who underwent this regimen for over a year [119].

There were two follow-up reports [120,121] that detailed the long-term outcomes of intranasal POH in a cohort of 198 recurrent patients, including 16 with anaplastic oligodendroglioma, 27 with grade III astrocytoma, and 155 with recurrent GB. POH was diluted in mineral water (pH > 7) and administered 4 times per day via nebulizer. The initial dose was 67 mg (268 mg/day), but was escalated to 133 mg (533 mg/day) based on its low toxicity profile. Overall survival was compared to histological classification, extent of peritumoral edema, topography, and tumor size. The results revealed that after 4 years of continuous, exclusive POH treatment, 19% of these patients remained under clinical remission, while side effects of this regimen were almost non-existent. This latter aspect, in combination with ease of self-administration and portability of the lightweight nebulizer, resulted in very high (>95%) patient compliance [121].

**Figure 3:**
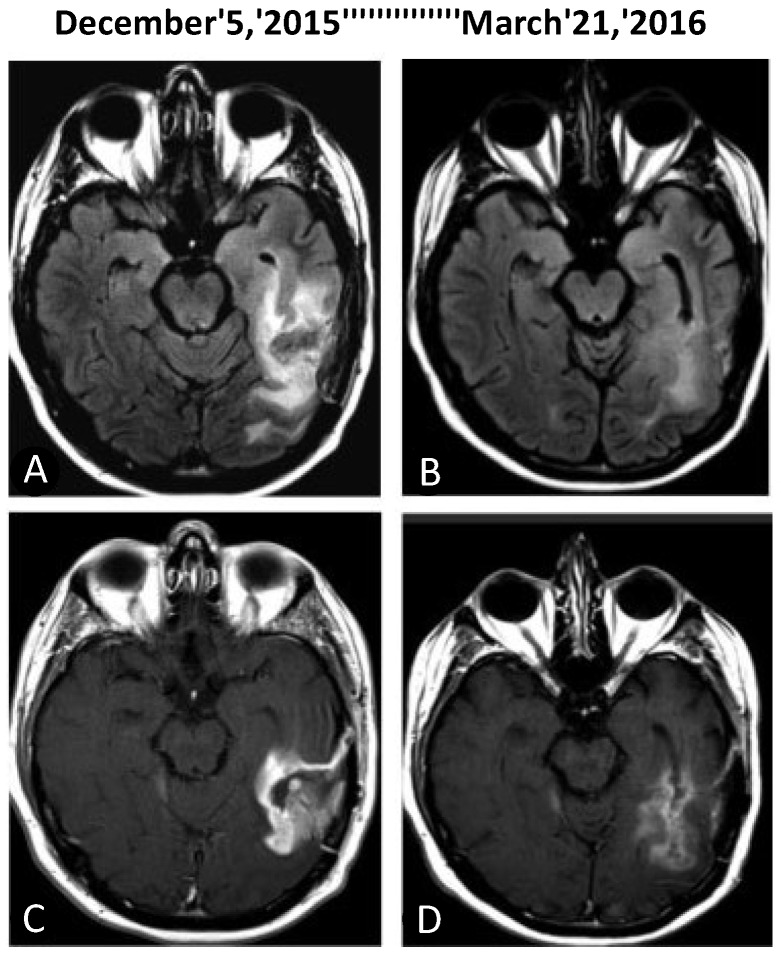
Effect of KD concomitant with inhalational POH in a patient with recurrent GB. Representative brain MRI scans are shown from before the onset of treatment (left panels) and after 3 months of treatment (right panels). (**A**,**B**) MRI axial fluid-attenuated inversion recovery (FLAIR); (**C**,**D**) T1-weighted image (T1W) with contrast. Note marked reduction of peritumoral edema and tumor size after treatment.

Collectively, these studies presented intranasal POH as a safe, non-invasive treatment option with activity in patients with recurrent malignant glioma [120,121]. Compared to those trials with oral POH described above, the inhalational dose of POH was substantially smaller: the highest dose used, 533 mg/day intranasal POH (equivalent to 271 mg/m^2^), was 18-fold lower than the standard daily dose (4800 g/m^2^) used in phase 2 trials of oral POH [113,114,115,116,117] (Table 1). It was noted that continuous dosing was essential, as patients who discontinued intranasal POH were prone to relapse (see example in Figure 3). As of 2018, there are patients who have been using this regimen for 8 years uninterrupted, pointing to the possibility that intranasal POH perhaps has the potential to convert malignant glioma from a deadly to a chronic disease in at least some of the patients.

Based in large part on these Brazilian studies, a similar clinical trial was initiated in the U.S. in 2016, and as of 2018 is recruiting patients with recurrent glioblastoma (grade IV glioma). It represents the first clinical trial of intranasal POH in the U.S. (ClinicalTrials.gov identifier: NCT02704858). It uses NEO100, which is a synthetic, highly pure (>99%) version of POH that is manufactured under good laboratory practice (GMP) conditions. This multicenter study is designed as a phase 1/2a trial, where phase 1 evaluates four escalating dosages for tolerability (384 mg, 576 mg, 768 mg, and 1152 mg per day, distributed over 4 equal doses and administered using a nebulizer and nasal mask). The maximum tolerated dose (MTD) will be extended in the 2a phase for a total of 25 patients; if MTD is not reached in phase 1, the highest evaluated dose (1152 mg) will be used in 2a (Table 1). The primary objectives of phase 1 include the determination of safety and tolerability of intranasal NEO100 in patients with recurrent (or progressive) grade IV glioma, and the identification of the MTD. The primary objective of phase 2a is determination of progression-free survival rate at 6 months. Altogether, this study represents a key step in the development of NEO100 as a novel, intranasally administered therapy for malignant glioma patients, who have become unresponsive to other types of therapy and face dismal prognosis. First results may become available in 2020. 

### 4.5. Intranasal POH in Combination

In view of the generally promising results obtained with intranasal POH monotherapy in patients with malignant glioma, there are efforts to explore intranasal POH in combination with other types of intervention. One such initiative examines combination with a ketogenic diet (KD), as there are indications that adjuvant KD might benefit certain types of cancer therapy [122,123,124]. Typically, a KD consists of high amounts of fat, moderate amounts of protein, and quite low amounts of carbohydrates, aimed at promoting a specific metabolic state that is characterized by low blood sugar levels and increased amounts of fat-derived ketone bodies. In the context of cancer therapy, the concept of KD is viewed as a metabolic therapy, rather than a dietary approach [125]. This model proposes that cancer cells that depend on increased sugar consumption (Warburg effect [126]) will be starved in the absence of plentiful carbohydrates, and thereby sensitized to concomitantly applied cancer therapeutic agents [122,123,125].

A recently published case study [127] reported on a patient with recurrent GB who responded favorably to KD combined with intranasal POH. This patient had undergone surgery, followed by standard chemoradiation (6 weeks of radiation plus temozolomide), followed by 3 cycles of adjuvant temozolomide. However, eight months later, the tumor had returned, and this time was chemoresistant, i.e., there was no reduction in tumor size in response to additional temozolomide. The patient was started on a KD, concomitant with 4 times daily inhalational POH (220 mg/day). Three months later, an MRI scan revealed significant reduction of the enhancing lesion. At the same time, there was some weight loss, along with a small increase in muscle mass. It was concluded that this therapeutic regimen was very well tolerated and appeared to have therapeutic benefit [127].

This encouraging result was followed by a larger study [128] with 32 recurrent malignant glioma patients divided into 2 groups, either on standard diet or on KD, along with intranasal POH for 3 months. Not all patients survived this time period, but the results for the survivors showed 7 of 9 patients with partial response, 1/9 with stable disease, and 1/9 with progressive disease in the group of patients adhering to KD. In comparison, data for patients with standard diet showed 2/8 partial response, 2/8 stable disease, and 4/8 with progressive disease. While these numbers were too small to establish statistical significance, there was a clear trend toward benefit for addition of KD. An example is shown in Figure 3. 

While the molecular mechanisms underlying potential synergy of POH and KD have not yet been investigated, one could hypothesize that cellular stress mechanisms, in particular the endoplasmic reticulum (ER) stress response, may play a critical role. The ER stress response represents a bifunctional cellular process that aims to adapt cells to stressful conditions, or, if the stressful impact is not manageable, triggers apoptotic cell death [129,130]. In the case of tumor cells, such stressor may include hypoxia, hypoglycemia, oxidative stress, and acidification, which result in continuous engagement of this stress response. While this chronic activation supports tumor cell survival under adverse microenvironmental conditions, it may also reveal an Achilles’ heel, because such “stressed out” cells are less able to neutralize additional taxing inputs, such as drug treatments [131,132].

The ER stress response system has been recognized as a possible therapeutic target in GB [133,134], which is of interest in the above context. POH has been shown to aggravate ER stress in GB cells in vitro, which at least in part contributes to subsequent cell death [73]. Similarly, chronic hypoglycemia experienced by tumor cells is expected to be exacerbated under conditions of KD [123,135,136]. One can thus hypothesize that the combined effects of POH-induced stress and KD-induced hypoglycemia result in aggravated ER stress that is severe enough to effectively trigger apoptosis in tumor cells. The validation of this concept will have to await the availability of surgical brain tumor samples obtained from patients undergoing this treatment, which can be subjected to immunohistochemistry for typical pro-apoptotic ER stress markers, such as CHOP (also called GADD153) [137].

### 4.6. Exploration of Novel Therapeutic Applications of POH

In the preclinical domain of POH research, a few recent studies have explored novel principles of POH use for therapeutic purposes, in particular in the context of improved brain targeting toward greater intracranial therapeutic activity. While not all of them involve the intranasal mode of delivery, they will be briefly introduced below.

#### 4.6.1. POH as a Vehicle for Binary Drug Delivery

One study [138] investigated whether the amphipathic features of POH could be harnessed to provide carrier function, with the objective to co-deliver other therapeutics into the brain via intranasal delivery. To establish proof of principle, the authors used an FDA-approved therapeutic that was well known to cross the BBB very poorly. This agent was bortezomib (Velcade^®^), which is widely used for the treatment of multiple myeloma patients [139]. Preclinical studies with cell lines and respective mouse models had shown that bortezomib was also effective against GB. Importantly, however, while intravenous bortezomib was active against subcutaneously implanted GB cells, it did not exert activity against orthotopically implanted GB cells, i.e., tumor cells located behind the BBB were protected from this therapy. On the other hand, when bortezomib was delivered via catheter directly into the brain, potent anticancer activity in the brain could be achieved [138,140,141,142].

In an effort to avoid the invasive aspect of catheter delivery, the authors tested whether a simple mix of bortezomib with POH (as NEO100), administered intranasally to animal tumor models, could exert therapeutic activity against orthotopic GB. This was indeed the case [138]. Brain tumor-bearing mice survived significantly longer when they received bortezomib formulated with NEO100, as compared to mice that received bortezomib in the absence of NEO100 or mice that received NEO100 alone. Moreover, bortezomib concentrations in the brain were substantially higher when this drug was co-administered with NEO100. Clearly, intranasal administration provided the decisive advantage, because intravenous delivery of bortezomib, with or without NEO100, was ineffective. Altogether, this study provided proof of principle that a POH/NEO100-based formulation can enable non-invasive, therapeutically effective brain delivery of an otherwise non-BBB-permeable pharmaceutical agent [138].

#### 4.6.2. POH as a Covalent Modification of Established Drugs

A flurry of in vitro and in vivo studies from the Chen lab at the University of Southern California [143,144,145,146,147,148] characterized a novel chemical composition, where POH was covalently conjugated to temozolomide (Temodar^®^; Temodal^®^), the current gold standard of chemotherapeutic care for malignant glioma. This new molecule, variously called TMZ-POH or NEO212, had emerged from in silico analysis based on a BBB penetration analysis software [34]. While computer modeling predicted enhanced BBB penetration by NEO212 as compared to the unconjugated temozolomide molecule, detailed studies in cell lines and mouse tumor models revealed additional physiochemical features that are expected to prove beneficial in future clinical trials.

Temozolomide, an alkylating agent, exerts its anticancer activity primarily via its ability to methylate the O6-position of guanine [149]. Importantly, this activity has been preserved in NEO212 at increased potency. The underlying mechanism of enhanced DNA methylation has not yet been completely validated, although it appears that greater cell entry of NEO212 over temozolomide may play a role [143]. In addition, after oral delivery to rodents, NEO212 penetrated the BBB significantly more effectively than temozolomide, as had been predicted by in silico modeling. Several independent animal tumor models consistently demonstrated superior therapeutic efficacy of NEO212 over temozolomide, including orthotopic models of GB, intracranial breast cancer metastases models, and tumor stem cell models with the use of primary, patient-derived GB tissue [143,146,147,148]. Intriguingly, in all cases the beneficial activity of NEO212 was greater than the sum of its parts: merely mixing POH with temozolomide in vitro, or co-delivering POH with temozolomide in vivo, was unable to mimic the significantly greater anticancer activity of the conjugated NEO212 molecule.

Altogether, NEO212 serves as a first-in-class compound where brain entry and intracranial activity of an existing pharmaceutical agent has been enhanced by covalent linkage to POH. Toxicological studies indicated that NEO212 was well tolerated after oral delivery [143,144,145], and activities are under way to obtain investigational new drug (IND) approval from the U.S. Food & Drug Administration to move NEO212 into clinical testing. Based on its excellent BBB-penetrating characteristics, it is expected that oral dosing will represent the most appropriate mode of drug delivery for GB patients. 

## 5. Conclusions and Outlook

The use of intranasal NEO100 (i.e., clinical-grade POH) as an agent to treat a neurological disease (e.g., GB) is unique in that it demonstrates the feasibility of nasal brain delivery via the cranial nerves (I, V), thereby avoiding the obstacle presented by the BBB. This delivery method also reduces exposure through the systemic circulation, by that minimizing the systemic side effects. Once the cranial nerve brain delivery method has been fully validated, it may be employed for delivery of other pharmacologic agents aimed not only at primary brain cancers, but also at secondary brain malignancies/metastases derived from systemic tumors, as well as different neurological diseases, including Parkinson’s, stroke, and Alzheimer’s. The era of treating neurological disorders effectively is hinged on better and more efficient delivery to the brain. While nasal brain delivery may not be the only route for effective drug delivery, it is non-invasive and very well tolerated by the patients. It is our premise that the time will come when nasal brain delivery will not only be effective, but generally accepted and routinely used.

## Figures and Tables

**Table 1:** Efficacy studies with perillyl alcohol.

Study Phase (Duration)	Patients	Dosing	Daily Doses (mg/m^2^/day)	N	Published Results
Phase 2 (6-month, daily dosing)	Ovarian cancer	Oral (qid)	4800 −6000	20	[114]
Phase 2 (6-month, daily dosing)	Prostate cancer	Oral (qid)	4800	15	[115]
Phase 2 (6-month, daily dosing)	Breast cancer	Oral (qid)	4800 −6000	14	[113]
Phase 2 (6-month, daily dosing)	Colorectal cancer	Oral (qid)	4800 −6400	27	[116]
Phase 2 (15-day pilot study)	Pancreatic cancer	Oral (qid)	4800	8	[117]
Phase 2 (long-term daily dosing)	Brain cancer	Intranasal (qid)	112 −271	>250	[118,119,120,121]
Phase 1/2a (NEO100) (long-term daily dosing)	Recurrent GB	Intranasal (qid)	195 −586	25 (Phase 2)	N/A

For qid dosing, the dosing intervals are scheduled at least 4 h apart (evenly spread throughout the day).

## Abbreviations

BBB	Blood-brain barrier
CHOP	CCAAT/Enhancer-binding protein homologous protein
CNS	Central nervous system
CSF	Cerebrospinal fluid
ER	Endoplasmic reticulum
FPTase	Farnesyl-protein transferase
GB	Glioblastoma (multiforme) (see ref. [150] for details)
GGPTase	Geranylgeranyl-protein transferase
GRP78	Glucose-regulated protein of 78 kDa
IC50	Inhibitory concentration at 50% effect
IUPAC	International Union of Pure and Applied Chemistry
IN	Intranasal
JNK	c-Jun N-terminal kinase
KD	Ketogenic diet
MRI	Magnetic resonance imaging
MTD	Maximum tolerated dose
Na/K-ATPase	Sodium/potassium-adenosine triphosphatase
NEO100	Clinical-grade, highly pure perillyl alcohol
POH	Perillyl alcohol
RT	Radiotherapy
TMZ	Temozolomide
